# Mucinous cystic neoplasm of the liver mimicking a hydatid cyst

**DOI:** 10.1002/ccr3.3575

**Published:** 2020-11-23

**Authors:** Faten Limaiem, Saâdia Bouraoui

**Affiliations:** ^1^ Tunis Faculty of Medicine University of Tunis El Manar Tunis Tunisia; ^2^ Department of Pathology University Hospital Mongi Slim La Marsa La Marsa Tunisia

**Keywords:** cyst, liver, mucinous cystadenoma; pathology, neoplasm

## Abstract

Misdiagnosis of mucinous cystic neoplasms of the liver may have serious consequences due to their premalignant potential. A thorough understanding of their characteristic features and a high index of suspicion are mandatory (*Mod Pathol*, 24, 2011, and 1079).

## Graphical abstract

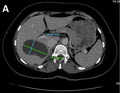

## 1 CLINICAL IMAGE

Mucinous cystic neoplasm of the liver is a rare slow‐growing lesion accounting for less than 5% of all hepatic cystic neoplasms.^1^, ^2^ Differential diagnosis from other cystic lesions remains challenging despite progress achieved in the radiological modalities. Only histopathological examination of the surgical specimen establishes with certainty in the diagnosis.^1^

**FIGURE 1:**
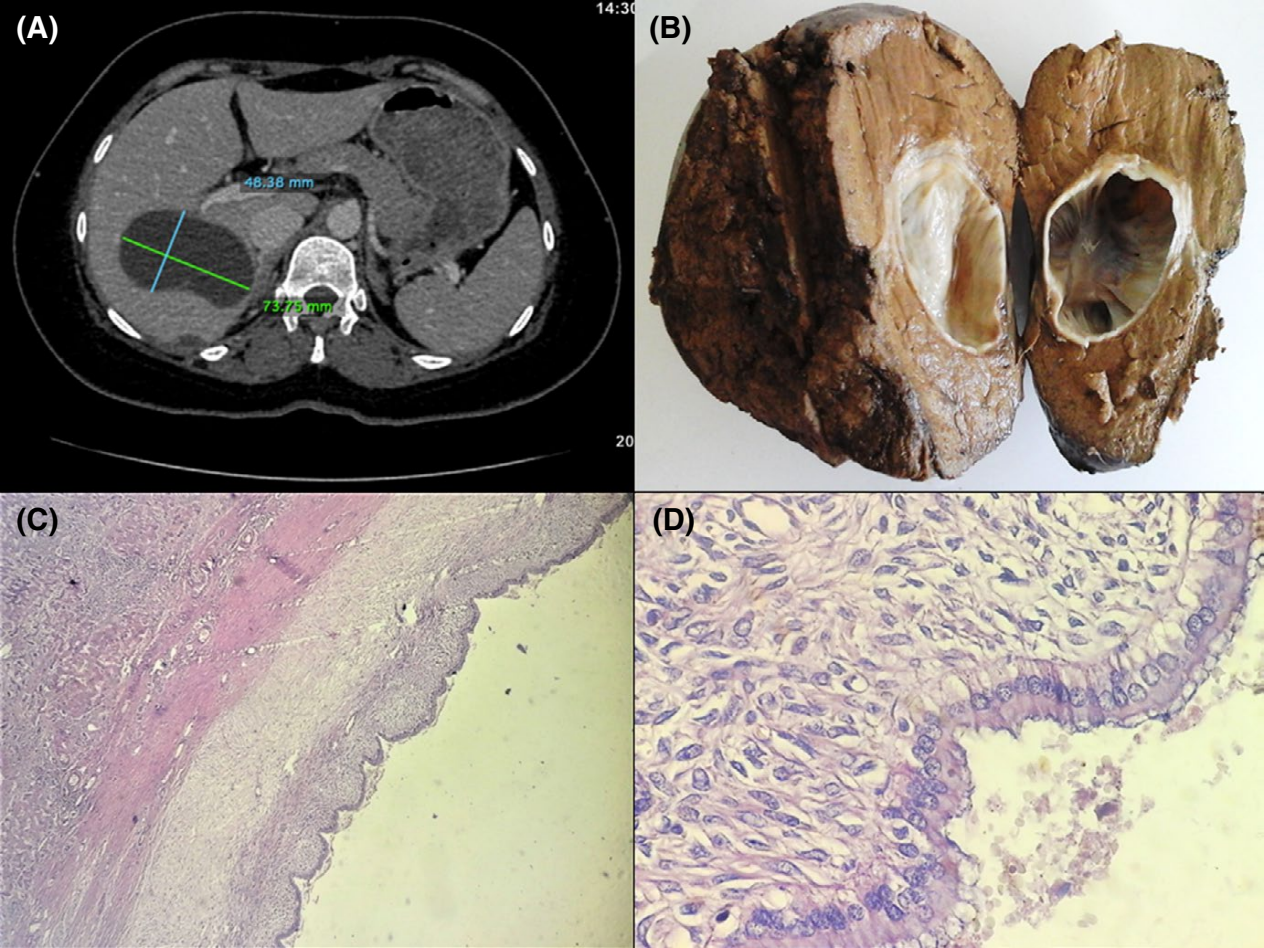
A, Axial CT scan demonstrating a unilocular cystic mass within the right hepatic lobe of the liver, measuring 73.75 × 48.38 mm. B, Macroscopic findings of mucinous cystadenoma. A unilocular well‐delineated cystic mass of the liver surrounded by a thick fibrous capsule. C, Solitary cystic lesion of the liver lined by simple epithelium overlying ovarian type stroma (Hematoxylin and eosin, magnification ×40). D, Histological examination of the hepatic cyst showing columnar biliary type epithelium overlying dense spindled ovarian type stroma (Hematoxylin and eosin, magnification ×400)

A 40‐year‐old female patient with a past medical history of hypothyroidism, presented with a 4‐month history of right upper quadrant pain and weight loss. Abdominal computed tomography scan revealed a 73.75 × 48.38 mm unilocular cystic mass in the right hepatic lobe suggestive of hydatid cyst (Figure 1A). Laboratory tests were within normal limits. Tumor markers (including CA19–9) and serum IgE level were not performed. Given her history of proximity to livestock in an endemic area, the diagnosis of a hydatid cyst was strongly favored. However, echinococcosis serology was negative. The patient underwent a right hepatectomy through a right subcostal incision. Grossly, the cystic mass was surrounded by a thick whitish capsule and contained a clear mucinous fluid (Figure 1B). Histological examination demonstrated a cystic lesion lined by a columnar biliary type epithelium overlying dense spindled ovarian‐type stroma (Figure 1C,D). There were signs of cytological atypia or mitoses. The final pathological diagnosis was mucinous biliary cystadenoma of the liver. The postoperative course was uneventful. At present, the patient is still being followed up.

## CONFLICT OF INTEREST

None declared.

## AUTHORS' CONTRIBUTIONS

FL: prepared, organized, wrote, and edited all aspects of the manuscript. She performed the gross and microscopic pathologic evaluation of the pathology specimen. She prepared all of the histology figures in the manuscript. She read, edited, and approved the final version of the manuscript. SB: participated in the drafting of the article and revising it critically for important intellectual content.

## ETHICAL APPROVAL

All procedures performed were in accordance with the ethical standards. The examination was made in accordance with the approved principles.

## Data Availability

In accordance with the DFG Guidelines on the Handling of Research Data, we will make all data available upon request.
